# Comparative Electrical and Photoresponse Investigation of the Au/PVA/n*-*Si (*MPS1*) and Au/(CdTe:PVA)/n-Si (*MPS2*) Photodiodes, Both in the Dark and under Illumination

**DOI:** 10.1021/acsomega.5c11375

**Published:** 2026-01-31

**Authors:** ÇiĞdem Şükriye Güçlü, Latif Barış Akman, ÇiĞdem Bilkan, Şemsettin Altındal

**Affiliations:** † Department of Physics, Faculty of Sciences, Gazi University, Ankara 06500, Türki̇ye; ‡ Department of Photonics, Faculty of Applied Sciences, Gazi University, Ankara 06500, Türki̇ye; § Tokat Gaziosmanpaşa University, Erbaa Vocational School of Health Services, Tokat 60500, Türki̇ye

## Abstract

In this current article, Au/n-Si (metal/semiconductor) *MS* devices with high-purity poly­(vinyl alcohol) (PVA) (99+% hydrolyzed) (*MPS1*) and Au/(CdTe:PVA)/n-Si (metal/polymer/semiconductor) (*MPS2*) are grown on the same n-type Si wafer by using the spin-coating technique and determine their effects on light sensitivity and basic electronic parameters like saturation current (*I*_0_), which is derived from the straight-line intercept of ln­(*I*) at *V* = 0, zero-bias barrier height (ϕ*_b_*_0_) at *V* = 0, ideality/quality factor (*n*), and both the shunt/series resistances (*R*_*s*_, *R*_sh_). For this purpose, the current–voltage (*I*–*V*) measurement is carried out over a wide voltage range (±4.5 V), both in the dark and under 100 mW/cm^2^ conditions. Experimental results showed that the calculated electrical parameters were highly dependent on light, the organic interlayere, and voltage. The profile of interface states *(N*_*ss*_) dependent on energy and voltage-dependent resistance *(R*_*i*_), which significantly limit the performance of the photodiode, wwas obtained from the Card-Rhoderick model and Ohm’s law for both before and after illumination, respectively. In addition, voltage-dependent curves of the photosensitivity, photoresponsivity (*R*), and specific detectivity (D*) were obtained for the 100 mW·cm^–2^ intensity. When the results obtained for *MPS1* and *MPS2* photodiodes were compared with each other and with the existing literature, it was observed that the *MPS2* exhibited significantly better performance. Therefore, it was shown that *MPS2* with the (CdTe:PVA) interlayer could be a good candidate for electrical, optical, and energy conversion applications.

## Graphical abstract

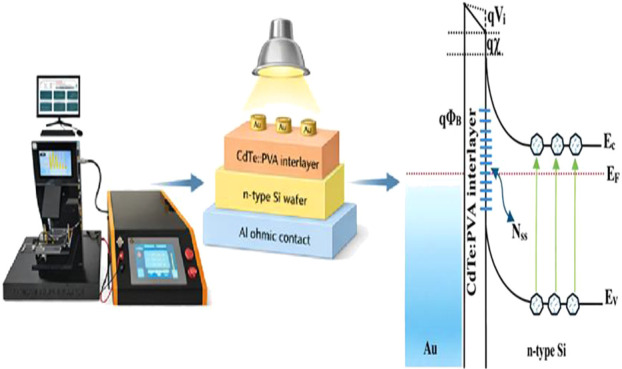

## 1 Introduction

The metal and metal/oxide-doped polymer interlayered metal/interlayer/semiconductor (*MIS*)- type structures, like Schottky/photodiodes (*SDs/PDs*), solar cells (*SCs*), and capacitors, have usually formed the basis of electronic and optoelectronic technologies over the last two decades.^1–7^ However, since there are many factors that determine the performance of these structures, either positively or negatively, there is no consensus on the current conduction/transport mechanisms (*CCMs/CTMs*), the nature of the barrier height (*BH*) formed at the metal–semiconductor interface, and the changes in their electrical and optical properties depending on light, temperature, and voltage. Since metal and semiconductors are contacted, the *BH* that forms at the *M/S* interface generally arises from the separation of charges at the junction. While electrons that are predominantly found on the front surface of an n-type semiconductor can diffuse to the front surface of the metal, leaving behind positively charged holes. When the system reaches thermal equilibrium, an internal electric field is generated from the semiconductor to the metal. The formation and nature of the *BH* at *M/S* interface are determined by the work functions of the chosen metal *(*ϕ_*m*_) and semiconductor *(*ϕ_*s*_), as well as the surface homogeneity of the semiconductor.^1–5^ To obtain rectifier behavior in an n-Si-based *SD*, the value of ϕ_*m*_ must be higher than ϕ_*s*_ and the transport of electrons from the n-Si to the metal leads to band bending in the conduction *(E*_*c*_) and valence band *(E*_*v*_) until their Fermi energy *(E*_*F*_) are aligned. Then, in thermal equilibrium, a *BH* forms at the *M/S* interface, which limits the movement of electrons/holes from the metal to the semiconductor or semiconductor to the metal.

In general, the use of a high-permittivity (high-ε*′*) interlayer between *M/S* interfaces can increase the quality of these devices with respect to a decrease of leakage/reverse current, *N*_*ss*_, *R*_*s*_, an increase in *BH, R*_sh_, and the rectification ratio (RR = *I*_*f*_ */I*_*r*_). Although traditional insulating interlayers, such as SiO_2_ and SnO_2_, are both stable and long lasting, they cannot reduce the performance-limiting factors, such as high leakage currents, *R*_*s*_, and *N*_*ss*_ in these devices to the desired level.^8–12^ Additionally, some organic interlayers usually have lower conductivity, lower dielectric properties, and reduced stability, but this problem can be overcome by using some doping metals (Zn, Ni, Mn, Co, Cu), graphene, and metal oxides (CuO, MnO, ZnO).^13–17^ In applications, unless specially fabricated, *MS*-type *SDs, PDs,* and *SCs* with and without an organic or oxide interlayer, both their *n* and *R_s_* values can deviate significantly from ideal conditions even at room temperature (*n* > 1, *R*_*s*_ > 0). This indicates that these devices show significant deviations from the standard or pure thermionic emission (*TE*) theory. Furthermore, very high-order energy levels *(N*_*ss*_) (>10^11^–10^14^ eV^–1^/cm^2^) can occur at the interlayer/semiconductor interface, with energies corresponding to the bandgap of the semiconductor.^18–23^ Moreover, these traps significantly affect the conduction mechanisms of these devices by capturing or releasing very high amounts of electrons under some external factors such as light and temperature.

There are similar studies in the literature on the electrical and optoelectrical characteristics of *MS*-type *SDs* with/without an interlayer.^23–30^ For instance, Ata et al.^23^ showed that introducing an MWCNT-doped PVA-B­(OH)_3_ layer in Au/n-Si *SDs* results in illumination-dependent variations in *BH*, *n*, and photocurrent, confirming their effective photosensing capability. Likewise, Surya Reddy et al.^24^ found that Au/V_2_O_5_/InP *MIS* diodes display systematic changes in key parameters with light intensity, demonstrating the photoactive contribution of the V_2_O_5_ interlayer. On the theoretical side, Di Bartolomeo et al.^25^ proposed a Landauer-based description of Schottky transport that captures tunneling effects and aligns well with experimental observations. Moreover, Beneldjemoui et al.^26^ reported that adding an ultrathin δ-GaN layer improves interface passivation in Au/δ-GaN/n-GaAs diodes, enhancing *BH* and producing a pronounced UV-visible photoresponse. Together, these findings underscore the importance of interface modification and advanced transport models in optimizing Schottky photodetectors. Karataş et al.^27^ have also studied the electrical and optical properties of *MS-* and *MIS*-type *SDs*.^28–30^ They found that these parameters and charge transport mechanisms are a strong function of the applied bias voltage, illumination, interfacial layer, and surface states or traps. To summarize, today, scientists, such as physicists, chemists, electronics engineers, and materials scientists have two main goals: one is to improve performance, and the other is to reduce production costs. This problem can be an obstacle to developing a new generation of interfacial layered materials that are both simple and fast to create and inexpensive. In other words, instead of traditional oxide layers, inexpensive, flexible, high-strength, and high-dielectric pure organic materials—either metal or metal oxide-doped—can be used.^22–26^

Therefore, in this manuscript, polyvinyl alcohol was chosen and doped with CdTe due to its some important features like direct bandgap, high absorption coefficient, and the creation of a high-mobility lifetime for electronic charges (electrons and holes), such as CdTe additive to the polymer, placed at *M–S* interface, and also leads to an increase in the performance of the device by passivating many unwanted interface states on the semiconductor surface and reducing both series resistance and leakage current.^6–10^ All findings indicate that the (CdTe:PVA) at the junction interface leads to a significant increase in both the illumination sensitivity and the performance of the *MS*-type *SDs* with respect to low *R*_*s*_, *N*_*ss*_, leakage current, and higher RR, *R*_sh_, and *BH* values, respectively.

## 2 Experimental Details

In this work, the (Cd­(CH_3_COO)_2_) and (Na_2_TeO_3_) precursors with 99% purity were provided by Merck Co. to grow CdTe nanostructures. After that, they were cleaned/filtered and then heated at 40 °C for 45 h. Au/(n-Si) (*MS*) devices with (pure-PVA) and (CdTe:PVA) thin interlayers (*MPS1, MPS2*) were fabricated on the n-type Si with P-doped single crystal in the same conditions, which has a <100> float zone, at about 300 μm thickness and 5 Ωcm resistivity. First, the wafer was cleaned by using acetone, methanol, H_2_O_2_, NH_4_OH, and HF solutions in an ultrasonic cleaner/bath to pick off the natural SiO_2_ and dried with nitrogen gas. Second, high-purity (99.999%) Al was evaporated onto the rear side of the wafer at 10^–6^ Torr and heated at 500 °C for 5 min to perform a low-resistivity contact. In this study, the PVA used, with *M_w_* 146,000–186,000 and 99+% hydrolyzed, was purchased from Aldrich-Chemistry company. Third, the (CdTe-PVA) composite was formed on the front of the n-Si substrate by the spin-coated technique. This technique is widely used to grow a thin interfacial film at the *M/S* interface as an interfacial layer, and its ability to produce highly uniform, smooth, and thickness-controlled films. Moreover, it offers some advantages, including low processing cost, operational simplicity, and suitability for a broad range of material systems, without the need for vacuum-based environments. Finally, high-purity Au (99.999%) rectifier contacts were also evaporated onto the polymer interlayer. The *I–V–P* measurements were done by Fytronix Co at RT. The schematic diagram of the *MPS PDs* and the measurement *I–V* system are represented in Figure 1a, and their energy diagram is given in Figure 1b, respectively.

**Figure 1:**
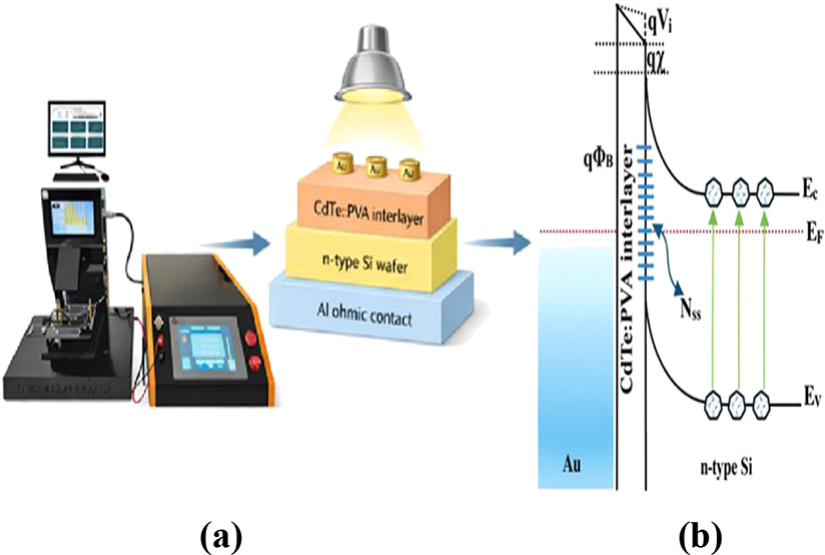
(a) Schematic representation of the *PDs* and (b) their energy diagram.

## 3 Results and Discussion

The ln­(*I*) vs *V* curves of the *MPS1/MPS2* Schottky-type *PDs* in the dark and under 100 mW·cm^–2^ between ±4.5 V are represented in Figure 2a and b, respectively. The ln­(*I*) vs *V* plots have a rectified behavior. While the value of the current shows a good rectifier feature at intermediate voltages, it deviates from linearity at high bias voltages because of the *R*_*s*_ and interlayer effects. Because the applied voltage on the diode is shared between the depletion regime, *R*_*s*_, and the interfacial layer. Contrary, the non- or unsaturated current in the reverse bias voltages can be attributed to *R*_sh_, generation and recombination, and the image lowering of the *BH* at junction.^3–7^

**Figure 2:**
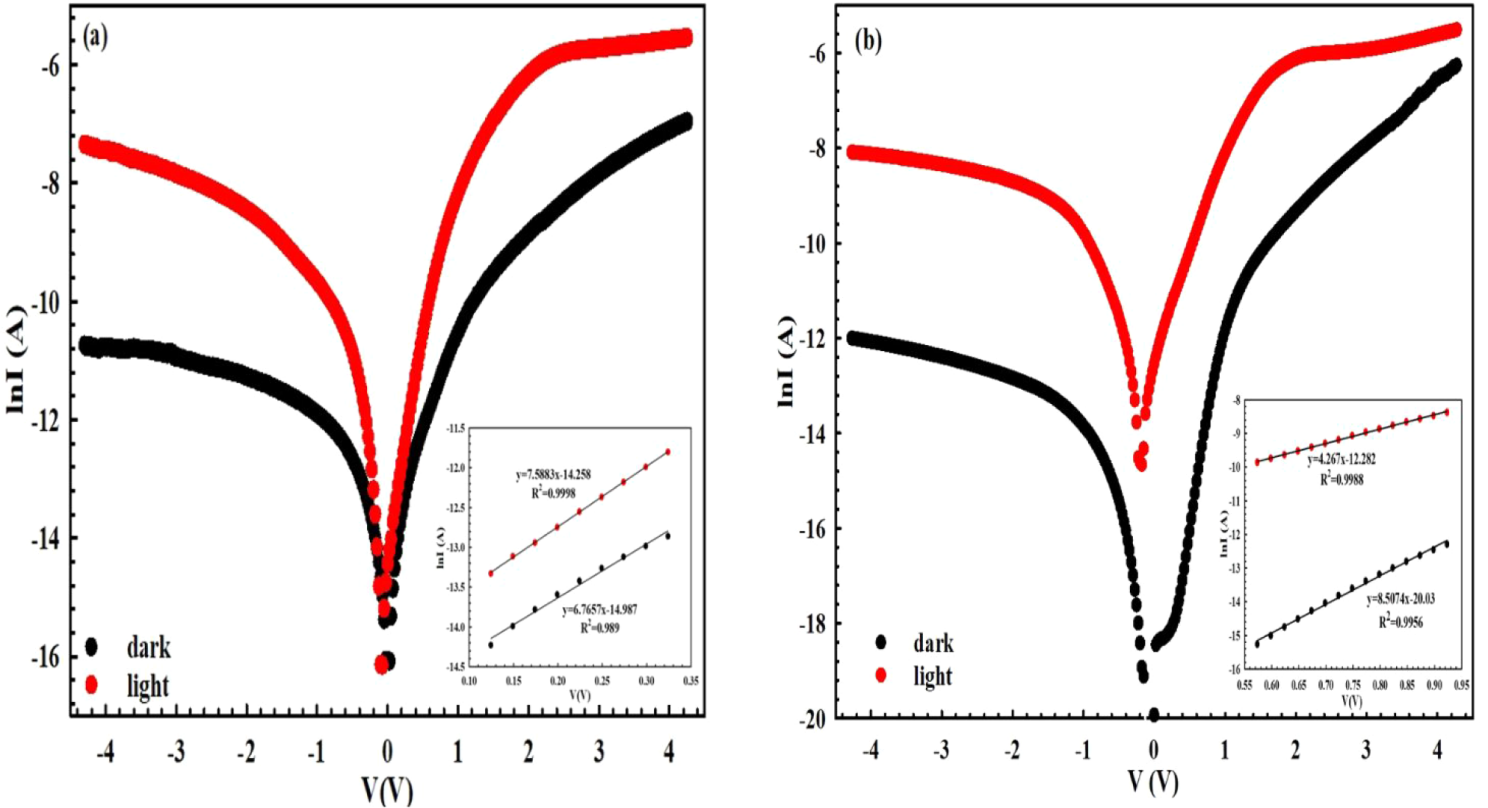
Semilogarithmic *I–V* curves: (a) *MPS1* and (b) *MPS2 PDs* in the dark/under illumination.

The *I* and *V* relation at *V* > (3*kT*/*q*), and the basic electronic features of these devices are calculated from the linear parts of the ln*(I)* vs *V* plot in the positive regime by using the TE model. According to TE, the current value for *MS-* and *MIS*-type *SDs* or *PDs* is given as follows:

1 $$I=\left(AA^{*}T^{2}\right)\exp\left(\frac{-q\Phi_{b0}}{kT}\right)\exp\left(\frac{q\left(V-IR_{s}\right)}{nkT}\right)$$

where *k* is the Boltzmann constant, *T* is the temperature in K, and the prefactor expression in front of the brackets is the saturation current (*I*_0_ or *I*_s_) and can be obtained from the interception point of the linear part of the ln*(I)–V* plot at *V* = 0.^1–3^ In addition, the quantities *A* and A* are the area of the Schottky contact (7.85 × 10^–3^ cm^2^) and the effective Richardson’s constant (=112 A/(cm·K)^2^ for n-Si), respectively. After obtaining the *I*_0_ value and by using *A*, the value of *Φ*_*b*0_ can be extracted as given in the following relation.^1,2^

2 $$\Phi_{b0}=\left(\frac{kT}{q}\right)\cdot \ln\left(\frac{AA^{*}T^{2}}{I_{0}}\right)$$

Schottky barrier diodes exhibit a parameter called the barrier height, which represents the energy difference between the metal’s Fermi level and the semiconductor’s conduction band edge at the interface. In essence, the barrier height determines the ease with which charge carriers can move across the metal–semiconductor junction, thereby governing the diode’s *I*–*V* characteristics, rectification behavior, and overall electronic performance. The ideality factor was also obtained from the linear regime of ln*(I)*–*V* curve as given by the following relations.^3–5^

3 $$n\left(V\right)=\frac{qV_{i}}{kT\left(\tan\theta \right)}=\frac{qV_{i}}{kT\left(d\ln\;I_{i}/dV_{i}\right)}=1+\frac{d_{i}}{\varepsilon_{i}}\left[\frac{\varepsilon_{s}}{W_{D}}+qN_{ss}\left(V_{i}\right)\right]$$

In eq 3, the quantities *d*_*i*_, ε_*i*_, ε_*s*_, ε_0_, and *W*_*D*_ are the interfacial layer thickness, permittivity of the semiconductor, permittivity of vacuum, and depletion layer width, respectively. The voltage dependence of resistance (ln­(*R*_*i*_) vs *V*) curve of this photodiode was also obtained from Ohm’s Law and is represented in Figure 3a,b. As seen in Figure 3, the *R*_*i*_ value becomes nearly constant at higher positive voltages and sufficiently lower negative voltages which correspond to the real *R*_*s*_ and *R*_sh_ values, respectively. The *I*_0_, Φ_*b*0_, *n, RR, R*_*s*_, and *R*_sh_ are tabulated in Table 1 for *MPS1*- and *MPS2*-type photodiodes.

**Figure 3:**
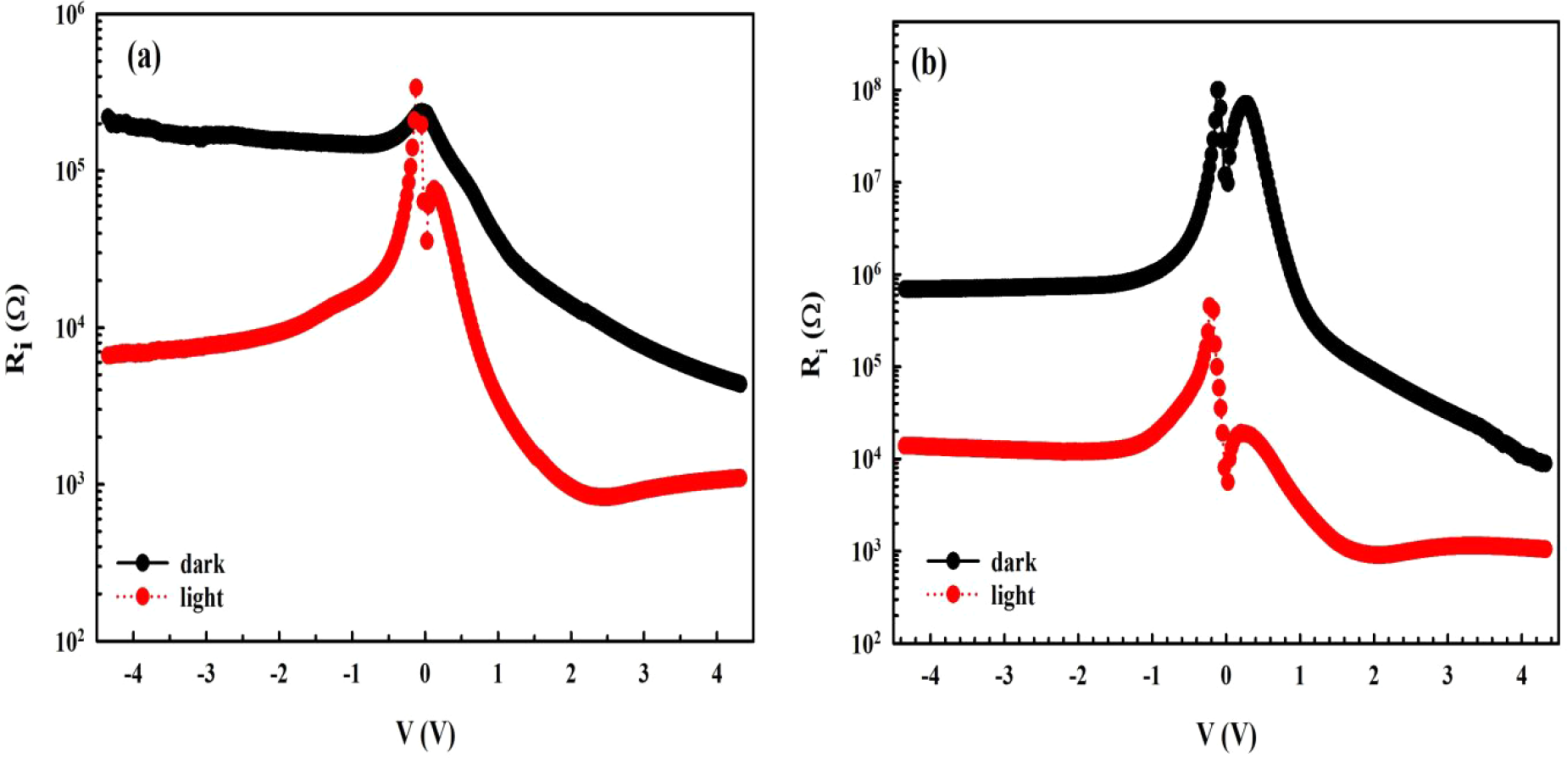
*R_s_–V* plot: (a) *MPS1-* and (b) *MPS2*-type *PDs* in the dark/illuminations.

**Table 1:** Fundamental Electronic Parameters of Fabricated *MPS1, MPS2 SDs* and Literature-Obtained Values from Various Calculation Methods: A) in the Dark and B) under 100 mW/cm^2^

	(A) In Dark												
	Samples	TE			Ohm’s Law			Cheung’s				Norde	
**This work**		***I***_**0**_ **(A)**	***n***	**Φ**_***b*0**_ **(eV)**	***R**_**s**_* **(Ω) (4.5 V)**	***R***_**sh**_ **(Ω) (−4.5 V)**	**RR (±4.5 V)**	***n* (d** ***V*** **/dln *I***)	***R***_***s***_ **(Ω) (d** ***V*** **/d ln *I***)	**Φ**_***b*0**_ **(eV)** ***H*** (I)	***R***_***s***_ **(Ω)** ***H*(I)**	**Φ**_***b*0**_ **(eV)**	***R**_**s**_* **(Ω)**
	**Au/PVA/n-Si**	5.33 × 10^–7^	5.27	0.52	4.11 × 10^3^	2.57 × 10^5^	61.88	8.53	7.13 × 10^3^	0.72	5.26 × 10^3^	0.65	2.47 × 10^4^
	**Au/CdTe:PVA/n-Si**	2.00 × 10^–9^	4.59	0.66	1.89 × 10^4^	6.79 × 10^5^	91.77	7.36	9.05 × 10^3^	0.70	1.05 × 10^4^	0.78	8.39 × 10^3^
Ref 20		***I***_**0**_ **(A)**	***n***	**Φ**_***b*0**_ **(eV)**	***R***_***s***_ **(kΩ) (3 V)**	***R***_**sh**_ (**MΩ) (−3 V)**	**RR (±4.5 V)**	***n* (d** ***V*** **/d ln *I***)	***R***_***s***_ **(kΩ) (d** ***V*** **/d ln *I***)	**Φ**_***b*0**_ **(eV)** ***H*** (I)	***R***_***s***_ **(kΩ)** ***H*** (I)	**Φ**_***b*0**_ **(eV)**	***R***_***s***_ **(kΩ)**
	**Au/n-Si**	7.89 × 10^–6^	7.65	0.596	1.60	0.209	13.1	8.61	1.35	0.46	1.31	0.60	1.07
	**Au/(Brushite + Monetite: PVC)/n-Si**	1.28 × 10^–9^	2.46	0.822	2.05	308.00	1.5 × 10^5^	6.55	1.58	0.70	1.17	0.862	46.62
Ref 21		***I***_**0**_ **(A)**	***n***	**Φ**_***b*0**_ **(eV)**	***R**_**s**_* **(Ω) (5 V)**	***R***_**sh**_ (**MΩ) (−5 V)**	**RR (±3 V)**	***n* (d** ***V*** **/d l**n ***I***)	***R***_***s***_ **(kΩ) (d** ***V*** **/d ln *I***)	**Φ**_***b*0**_ **(eV)** ***H*(I**)	***R***_***s***_ **(kΩ)** ***H*** (I)	**Φ**_***b*0**_ **(eV)**	***R***_***s***_ **(kΩ)**
	**Au/(Er**_**2**_ **O**_**3**_ **:PVC)/n-Si**	6.93 × 10^–10^	1.93	0.838	858.39	78.183		7.25	0.90	0.664	0.72	0.959	17.73
Ref 22		***I***_**0**_ **(pA)**	***n***	**Φ**_***b*0**_ **(eV)**	***R***_***s***_ **(Ω) (5 V)**	***R***_**sh**_ **(Ω) (−5 V)**	**RR (±4 V)**	***n* (d** ***V*** **/d ln *I***)	***R***_***s***_ **(Ω) (d** ***V*** **/d** **ln *I***)	**Φ**_***b*0**_ **(eV)** ***H*** (I)	***R***_***s***_ **(Ω)** ***H*(I)**	**Φ**_***b*0**_ **(eV)**	***R***_***s***_ **(Ω)**
	**Au/P(EHA)/n-Si**	8.3	4.15	0.92	1.84 × 10^5^	4.89 × 10^7^	2.70 × 10^2^	4.41	2.08 × 10^5^	0.97	1.63 × 10^5^	-	-
	**Au/P(EHA-** *co* **-AA)/n-Si**	97.10	7.09	0.88	1.37 × 10^4^	3.66 × 10^7^	1.83 × 10^3^	4.59	2.44 × 10^5^	0.92	1.37 × 10^5^	-	-
Ref 23		***I***_**0**_ **(A)**	***n***		***R***_***s***_ **(Ω) (2 V)**	***R***_**sh**_ (**MΩ) (−2 V)**	**RR (±2 V)**	***n* (d** ***V*** **/d ln *I***)	***R***_***s***_ **(Ω) (d** ***V*** **/d ln *I***)	**Φ**_***b*0**_ **(eV)** ***H*** (I)	***R***_***s***_ **(Ω)** ***H*(I)**	**Φ**_***b*0**_ **(eV)**	***R**_**s**_* **(Ω)**
	**Au/(MWCNT:PVA-B(OH)**_**3**_ **)/n-Si**	1.37 × 10^–9^	2.36	0.819	429.89	36	15.5	3.67	337.82	0.62	266.56	-	-
Ref 31		***I***_**0**_ **(**μ**A)**	***n***		***R**_**s**_* **(Ω) (3 V)**	***R***_**sh**_ **(Ω) (−3 V)**	**RR**	***n* (d** ***V*** **/d ln *I***)	***R***_***s***_ **(Ω) (d** ***V*** **/d ln *I***)	**Φ**_***b*0**_ **(eV) *H*(I)**	***R***_***s***_ **(Ω)** ***H*(I)**	**Φ**_***b*0**_ **(eV)**	***R***_***s***_ **(kΩ)**
	**Al/p-Si**	74.26	2.55	0.591	76.05	75.87	-	-	-	-	-	0.581	0.193
	**Al/(PVP:ZnTiO**_**3**_ **)/p-Si**	0.08	2.75	0.768	1040	9921	-	-	-	-	-	0.725	411.70
Ref 32		***I***_**0**_ **(A)**	***n***	**Φ**_***b*0**_ **(eV)**	***R***_***s***_ **(kΩ) (3.5 V)**	***R***_**sh**_ (**MΩ) (−3.5 V)**	**RR (±3.5 V)**	***n* (d** ***V*** **/d ln *I***)	***R***_***s***_ **(kΩ) (d** ***V*** **/d ln *I***)	**Φ**_***b*0**_ **(eV)** ***H*** (I)	***R***_***s***_ **(kΩ)** ***H*** (I)	**Φ**_***b*0**_ **(eV)**	***R**_**s**_* **(kΩ)**
	**Au/n-Si**	8.29 × 10^–6^	5.85	0.59	0.47	0.16	34	4.4	0.325	0.64	0.332	0.57	0.40
	**Au/PVC/n-Si**	1.12 × 10^–7^	3.98	0.7	1.02	0.46	455	5.77	0.41	0.75	0.475	0.72	1.68
	**Au/(PVC:Sm**_**2**_ **O**_**3**_ **)/n-Si**	7.58 × 10^–10^	2.27	0.84	0.35	1.39	3994	3.46	1.11	0.84	1.10	0.84	0.945
Ref 33		***I***_**0**_ **(A**)	***n***	**Φ**_***b*0**_ **(eV)**	***R***_***s***_ **(Ω)**	***R***_**sh**_ **(Ω)**	**RR**	***n* (d** ***V*** **/d** **ln *I***)	***R***_***s***_ **(Ω)** **(d** ***V*** **/d ln *I***)	**Φ**_***b*0**_ **(eV)** ***H*** (I)	***R**_**s**_* **(Ω) *H*(I)**	**Φ**_***b*0**_ **(eV)**	***R**_**s**_* **(Ω)**
	**Au/n-Ge**	-	1.25	0.62	-	-	-	1.97	3027	0.61	2974	0.63	2458
	**Au/MB/n-Ge**	-	1.30	0.63	-	-	-	2.19	38	0.62	37	0.63	2666
Ref 34		***I***_**0**_ **(A)**	***n***		***R***_***s***_ **(Ω)**	***R***_**sh**_ **(Ω)**	**RR**	***n* (d** ***V*** **/d ln *I***)	***R***_***s***_ **(MΩ) (d** ***V*** **/d ln *I***)	**Φ**_***b*0**_ **(eV)** ***H*** (I)	***R***_***s***_ **(MΩ) *H*(I)**	**Φ**_***b*0**_ **(eV)**	***R***_***s***_ **(MΩ)**
	**Au/n-InP**	2.76 × 10^–9^	1.94	0.74	-	-	-	2.01	30.56	0.75	32.52	0.76	3.50
	**Au/BST/n-InP**	5.01 × 10^–10^	2.05	0.83	-	-	-	2.11	100.35	0.84	110.17	0.83	593.42
Ref 35		***I***_**0**_ **(A)**	***n***		***R***_***s***_ **(Ω)**	***R***_**sh**_ **(Ω)**	**RR**	***n* (d** ***V*** **/d ln *I***)	***R***_***s***_ **(MΩ) (d** ***V*** **/d ln *I***)	**Φ**_***b*0**_ **(eV)** ***H*** (I)	***R_s_* (MΩ) *H*(I)**	**Φ**_***b*0**_ **(eV)**	***R***_***s***_ **(Ω)**
	**Ni/PSR/n-Si**	1.95 × 10^–10^	1.68	0.87	-	-	-	-	-	-	-	0.87	-
	**Ni/PSR/p-Si**	1.48 × 10^–8^	1.93	0.73	-	-	-	-	-	-	-	0.76	-

	(B) Under 100 mW/cm^2^												
	Interlayer	TE			Ohm’s law			Cheung’s				**Norde**	
**This work**		***I***_**0**_ **(A)**	***n***	**Φ**_***b*0**_ **(eV)**	***R***_***s***_ **(Ω) (4.5 V)**	***R***_**sh**_ **(Ω) (−4.5 V)**	**RR (±4.5 V)**	***n* (d** ***V*** **/d ln *I***)	***R***_***s***_ **(Ω) (d** ***V*** **/d ln *I***)	**Φ**_***b*0**_ **(eV) *H*(I)**	***R*_*s*_ (Ω)** ***H*(I)**	**Φ**_***b*0**_ **(eV)**	***R**_**s**_* **(Ω)**
	**Au/PVA/n-Si**	6.87 × 10^–7^	8.3	0.51	1.11 × 10^3^	6.82 × 10^3^	5.96	9.33	3.61 × 10^2^	0.60	2.84 × 10^2^	0.63	4.85 × 10^2^
	**Au/CdTe:PVA/n-Si**	4.59 × 10^–6^	9.14	0.46	1.02 × 10^3^	1.37 × 10^4^	13.67	9.15	2.48 × 10^2^	0.42	3.69 × 10^2^	0.59	2.13 × 10^2^
Ref 20		***I***_**0**_ **(A)**	***n***	**Φ**_***b*0**_ **(eV)**	***R***_***s***_ **(kΩ) (3 V)**	***R***_**sh**_ **(MΩ)** **(−3 V)**	**RR (±3 V)**	***n* (d** ***V*** **/d ln *I***)	***R***_***s***_ **(kΩ) (d** ***V*** **/dln *I***)	**Φ**_***b*0**_ **(eV) *H*(I)**	***R***_***s***_ **(kΩ)** ***H*** (I)	**Φ**_***b*0**_ **(eV)**	***R**_**s**_* **(kΩ)**
	**Au/n-Si**	1.83 × 10^–5^	8.77	0.57	0.63	0.122	19.5	9.74	0.34	0.42	0.37	0.59	0.30
	**Au/(Brushite + Monetite:PVC)/n-Si**	3.14 × 10^–8^	2.28	0.74	0.60	2.40	4 × 10^–3^	6.93	0.29	0.61	0.23	0.78	2.36
Ref 21		***I***_**0**_ **(A)**	***n***	**Φ**_***b*0**_ **(eV)**	***R***_***s***_ **(Ω) (5 V)**	***R***_**sh**_ **(MΩ)** **(−5 V)**	**RR**	***n* (d** ***V*** **/d ln *I***)	***R***_***s***_ **(kΩ) (d** *V* **/d ln *I***)	**Φ**_***b*0**_ **(eV) *H*(I)**	***R*_*s*_ (kΩ) *H*(I)**	**Φ**_***b*0**_ **(eV)**	***R***_***s***_ **(kΩ)**
	**Au/(Er**_**2**_ **O**_**3**_ **:PVC)/n-Si**	2.24 × 10^–9^	2.21	0.81	553.32	0.86	-	8.76	0.23	0.628	0.172	0.856	10.35
Ref 22		***I***_**0**_ **(nA)**	***n*n**	**Φ**_***b*0**_ **(eV)**	***R**_**s**_* **(Ω) (5 V)**	***R***_**sh**_ **(Ω) (−5 V)**	**RR**	***n* (d** ***V*** **/d ln *I***)	***R***_***s***_ **(kΩ) (d** ***V*** **/d ln** ***I***)	**Φ**_***b*0**_ **(eV) *H*(I)**	***R**_**s**_* **(kΩ)** ***H*** (I)	**Φ**_***b*0**_ **(eV)**	***R**_**s**_* **(Ω)**
	**Au/P(EHA)/n-Si**	0.42	4.6	0.81	8.22 × 10^3^	9.64 × 10^4^	-	5.58	7.21	0.77	6.16	-	-
	**Au/P(EHA-** *co* **-AA)/n-Si**	268	6.99	0.68	4.47 × 10^3^	5.34 × 10^4^	-	5.08	5.41	0.68	4.61	-	-
Ref 23		***I***_**0**_ **(A)**	***n***	**Φ**_***b*0**_ **(eV)**	***R***_***s***_ **(Ω) (2 V)**	***R***_**sh**_ **(Ω)** **(−2 V)**	**RR (±2 V)**	***n* (d** ***V*** **/d ln *I***)	***R**_**s**_* **(Ω) (d** ***V*** **/d ln *I***)	**Φ**_***b*0**_ **(eV) *H*(I)**	***R*_*s*_ (Ω)** ***H*(I)**	**Φ**_***b*0**_ **(eV)**	***R***_***s***_ **(Ω)**
	**Au/(MWCNT:PVA-B(OH)**_**3**_ **)/n-Si**	1.64 × 10^–9^	2.34	0.82	409.45	21	17.54	2.99	332.02	0.76	234.96	-	-
Ref 31		***I***_**0**_ **(A)**	***n***	**Φ**_***b*0**_ **(eV)**	***R***_***s***_ **(kΩ) (3 V)**	***R***_**sh**_ **(kΩ)** **(−3 V)**	**RR**	***n* (d** ***V*** **/d ln *I***)	***R***_***s***_ **(Ω) (d** ***V*** **/d** l**n *I***)	***Φ***_***b*0**_ **(eV) *H*(I)**	***R***_**s**_ **(Ω)** ***H*(I)**	***Φ***_***b*0**_ **(eV)**	***R***_***s***_ **(kΩ)**
	**Al/p-Si**	120.38	2.59	0.58	0.048	0.054	-	-	-	-	-	0.571	0.048
	**Al/PVP:ZnTiO**_**3**_ **/p-Si**	8.23	5.69	0.65	0.40	14.11	-	-	-	-	-	0.671	46.17
Ref 35		***I***_**0**_ **(A)**	***n***	**Φ**_***b*0**_ **(eV)**	***R***_***s***_ **(Ω)**	***R***_**sh**_ **(Ω)**	**RR**	***n* (d** ***V*** **/d ln *I***)	***R***_**s**_ **(Ω) (d** ***V*** **/d ln** ***I***)	**Φ**_***b*0**_ **(eV) *H*(I)**	***R***_***s***_ **(Ω)** ***H*(I)**	***Φ***_***b*0**_ **(eV)**	***R***_***s***_ **(Ω)**
	**Ni/PSR/n-Si**	-	1.98	0.84	-	-	-	-	-	-	-	0.84	-
	**Ni/PSR/p-Si**	-	-	-	-	-	-	-	-	-	-	-	-

The fundamental electronic parameters *(I*_0_, *n,* ϕ_*b*0_, *R*_*s*_, *R*_sh_) of the performed *MPS1-* and *MPS2*-type SBDs in the dark/under 100 mW/cm^2^ are given in Table 1A and B, respectively, and compared with similar structures which have been carried out by different researchers in recent years.^31–35^ When Table 1 is examined, the performance of the samples we prepared appears generally better than that of the others. This shows that the organic interfacial layer used (CdTe:PVA) considerably increases the quality of the Au/n-Si (*MS*) structure. To determine how these parameters change with the applied voltage as well as the illumination intensity, and how the calculation system used affects the results, these parameters were also calculated from the Cheung^36^ and Norde functions^37^ and are given in Table 1. As can be clearly seen in Table 1, there are some discrepancies between basic electrical parameters due to their *V* dependence and the nature of the calculation models, which correspond to different voltages. For instance, TE theory corresponds to the intermediate bias voltages, Cheung’s functions correspond to higher bias voltages, and the Norde function corresponds to lower bias voltages.

According to Cheung & Cheung, they observed a deviation from linear behavior at higher positive voltages in the ln*(I)*–*V* features, which can be explained by the presence of *R*_*s*_ and the interlayer. Because of this, the applied voltage onto *SDs* will be shared between them. For this region, the basic electrical parameters were also calculated by using the following relations, as given in Figures 4 and 5.^36^

4 $$\frac{dV}{d\;\ln\;I}=\left(\frac{nkT}{q}\right)+IR_{S}$$

5 $$H=V-n\cdot \left(\frac{kT}{q}\right)\ln\left(\frac{I}{AA^{*}T^{2}}\right)=n\Phi_{b}+IR_{s}$$

**Figure 4:**
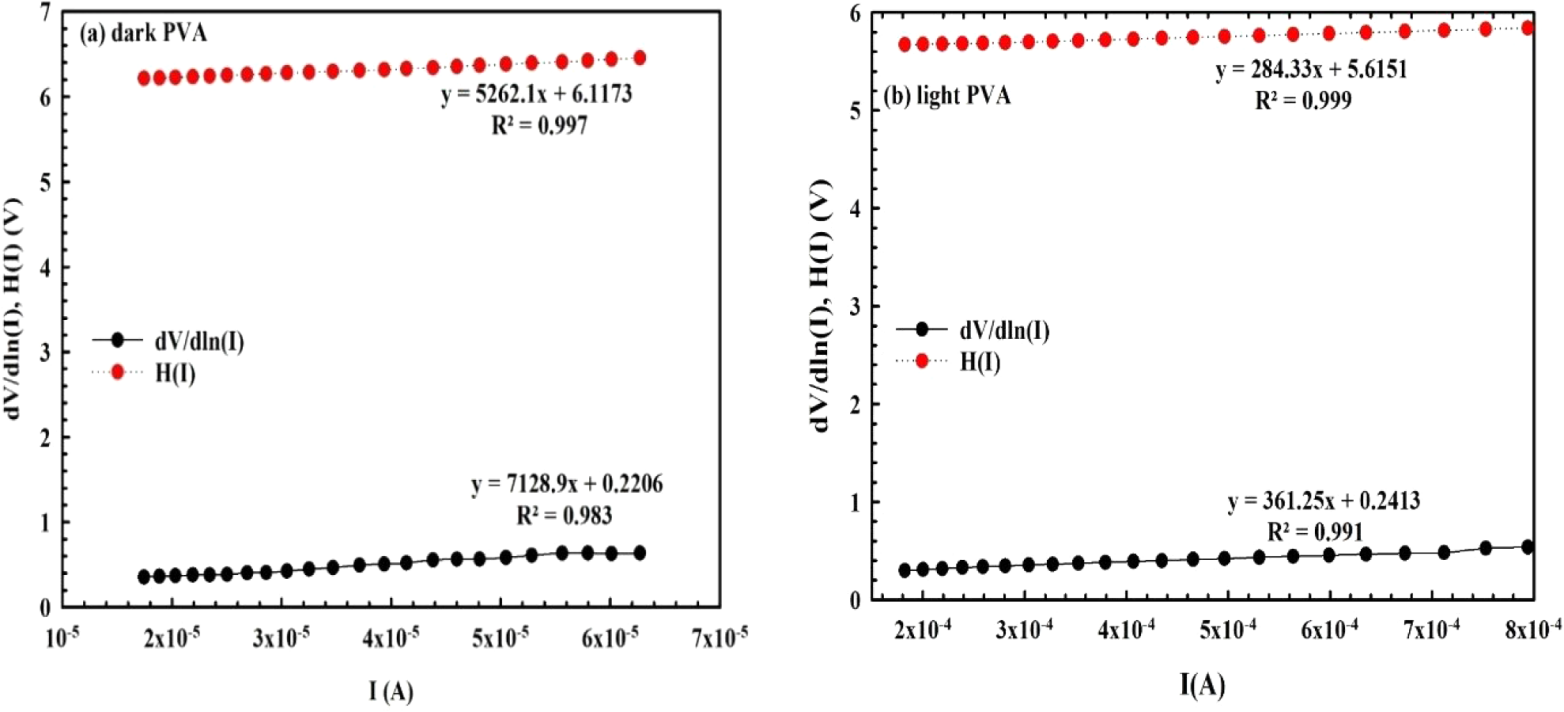
d*V*/d ln­(*I*) vs *I* and H­(*I*) vs *I*: (a) in the dark and (b) under illumination for *MPS1 SBD.*

**Figure 5:**
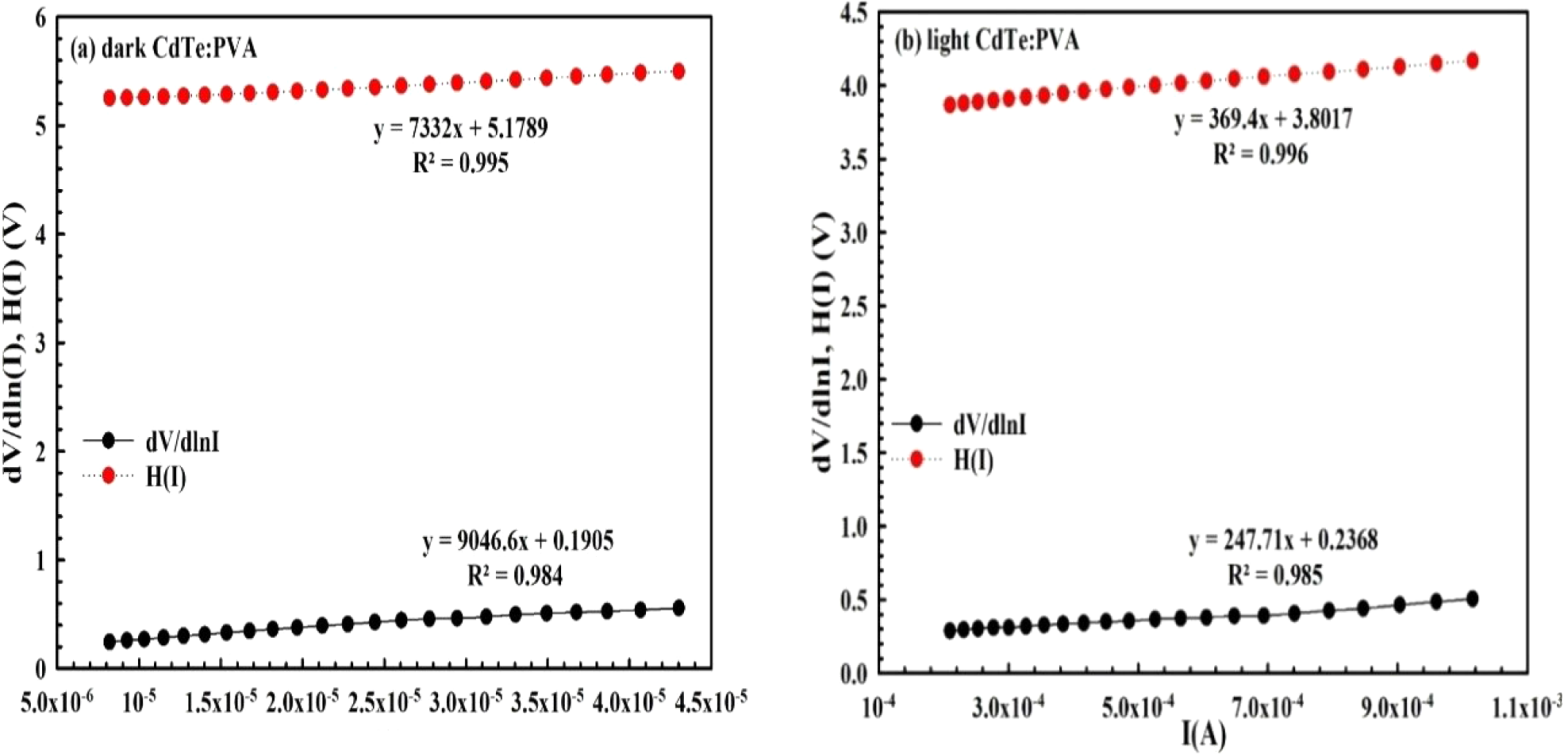
d*V*/d ln­(*I*) vs *I* and H­(*I*) vs *I*: (a) in the dark and (b) under illumination for *MPS2 SBD*.

As shown in these figures, the *n* and *R*_*s*_ value can be extracted from the intercept and slope of the d*V*/d ln­(*I*) vs *I* curve by using eq 4, respectively. The *BH* and *R*_*s*_ values (as a second way) can also be calculated from the intercept and slope of the *H–I* curve using eq 5, respectively. As can be clearly seen in Figures 4 and 5, both the d*V*/d ln­(*I*)–*I* and *H–I* plots show good linearity over a wide range of voltages and currents. If the semilogarithmic *I–V* curve does not have a good linear region, then both the accuracy/reliability of the calculated basic electrical parameters (*n,* ϕ_*b*_, *R*_*s*_) from its slope and intercept voltage at zero bias are questionable. In this case, Norde developed an eq 5 given below, based on TE theory when the value of *n* is considerably higher than unity.^30^

6 $$F\left(V\right)=\left(\frac{V_{i}}{\gamma }\right)-\left(\frac{kT}{q}\right)\left[\ln\left(\frac{I\left(V_{i}\right)}{AA^{*}T^{2}}\right)\right]$$

Here, γ is a constant and must be bigger than the *n* value to get a minimum point in the *F*(*V*)–*V* plot (Figure 6). As seen in Figure 6, the *F–V* plot has a distinctive minimum point for two types of *PDs,* and the corresponding voltage and current are called *V*_min_ and *I*_min_. After that, the values of Φ_*b*_ and *R*_*s*_ were obtained using the following relation and are tabulated in Table 1.^37^

**Figure 6:**
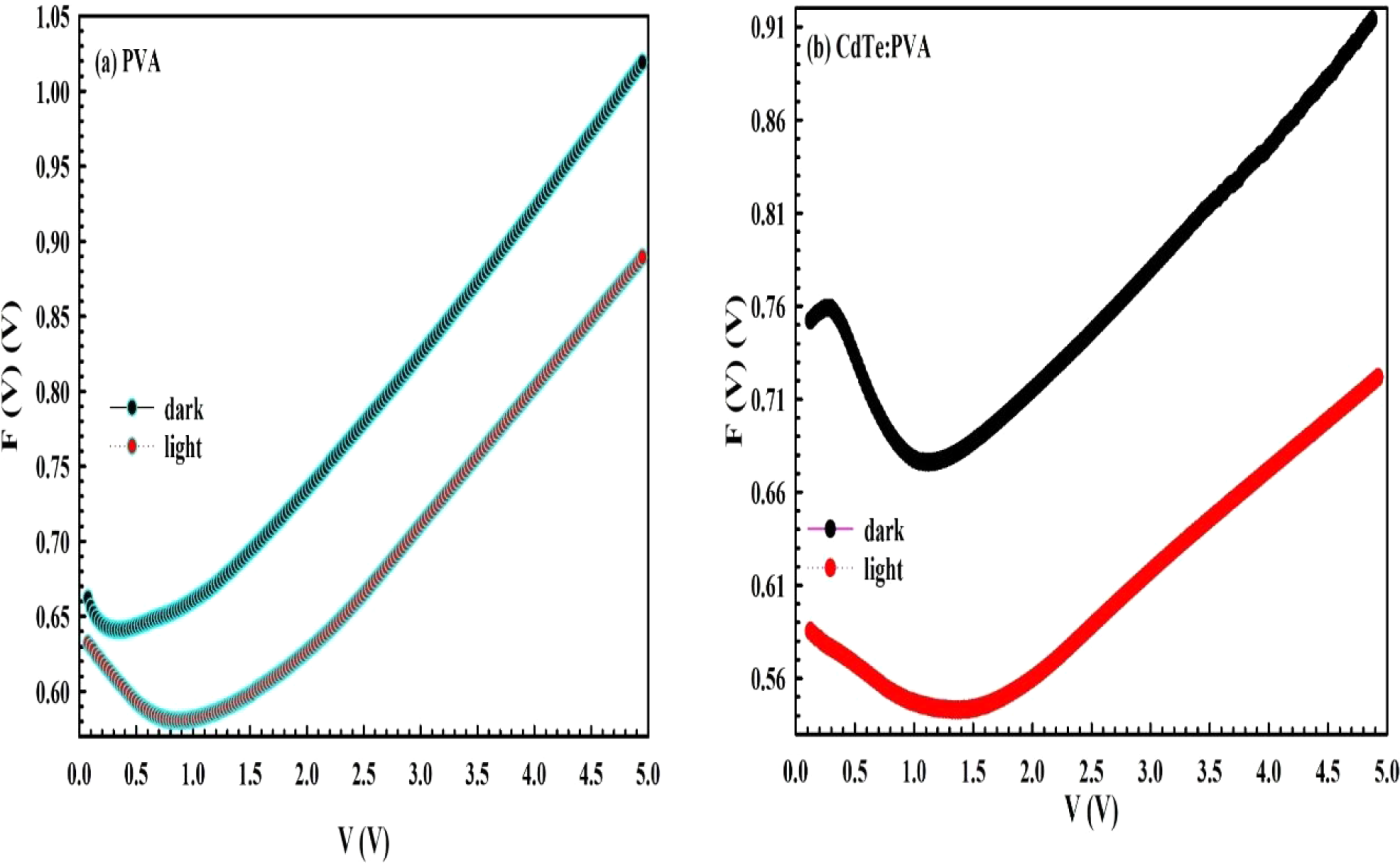
*F*(*V*) vs *V*: (a) for the *MPS1-* and (b) for the *MPS2*-type PDs.


7 $$\Phi_{b}=F\left(V_{\min}\right)+\frac{V_{\min}}{\gamma }-\left(\frac{kT}{q}\right)$$

8 $$R_{s}=\frac{kT\left(\gamma -n\right)}{qI_{\min}}$$

As can be seen from Figure 6, the F­(*V*)–*V* plot for these *PDs* has a clear minimum point at the moderate bias voltage in dark/illumination conditions. The magnitude and position of the minimum point of the *F–V* plot decrease under the illumination effec and shift toward higher positive voltages, respectively. Such features can be explained by the creation of electron and hole pairs, which produce an additional bias voltage. Electrons that gain enough energy under the illumination effect can excite from states to states or conduction bands, causing an increase in conductivity or a decrease in *R*_*s*_.

Thus, the Φ_*b*_ and *R*_*s*_ values of these *SBDs* are calculated from eqs 7 and 8 and Figure 6a,b by using the minimum point (*F*(*V*_min_)) and corresponding to the *V*_min_ and *I*_min_ points. The obtained values of Φ_*b*_ and *R*_*s*_ of these two diodes, as seen in Table 1, are compared with the TE model and Cheungs’ functions. As shown in Table 1, there are some differences between them due to the calculation models and voltage dependence. Because these calculation methods are valid in different applied voltage ranges. That is, the Norde function is usually valid at lower forward bias voltages, the TE theory is valid at moderate bias voltages or the linear part or ln*I*–*V* plot, and the Cheung functions are valid at enough higher voltages or when ln*I*–*V* plot deviates from the linear part.

To determine the light sensitivity of the prepared *MPS1* and *MPS2* Schottky-type photodiodes, the *V*-dependent profile of fundamental *PD* parameters, like photosensitivity (*S*), photoresponsivity (*R*), and photoelectricity (R*), are calculated from eq 7a–c for 100 mW/cm^2^ and are given in Figure 7a–c and Figure 8a–c, respectively. The photosensitivity of a photodiode is known as the ratio of its measurement of current in the dark to the photocurrent in the negative bias region rather than the forward bias region. Because in the reverse-bias condition, both the interior and applied electric fields at the junction have the same direction, the electric field is very strong, but under forward bias conditions, they have different directions, leading to a low electric field. Therefore, the created electron–hole pairs under illumination effect will be forced to move in different directions, giving a clear photocurrent in circuit.^38–42^

**Figure 7:**
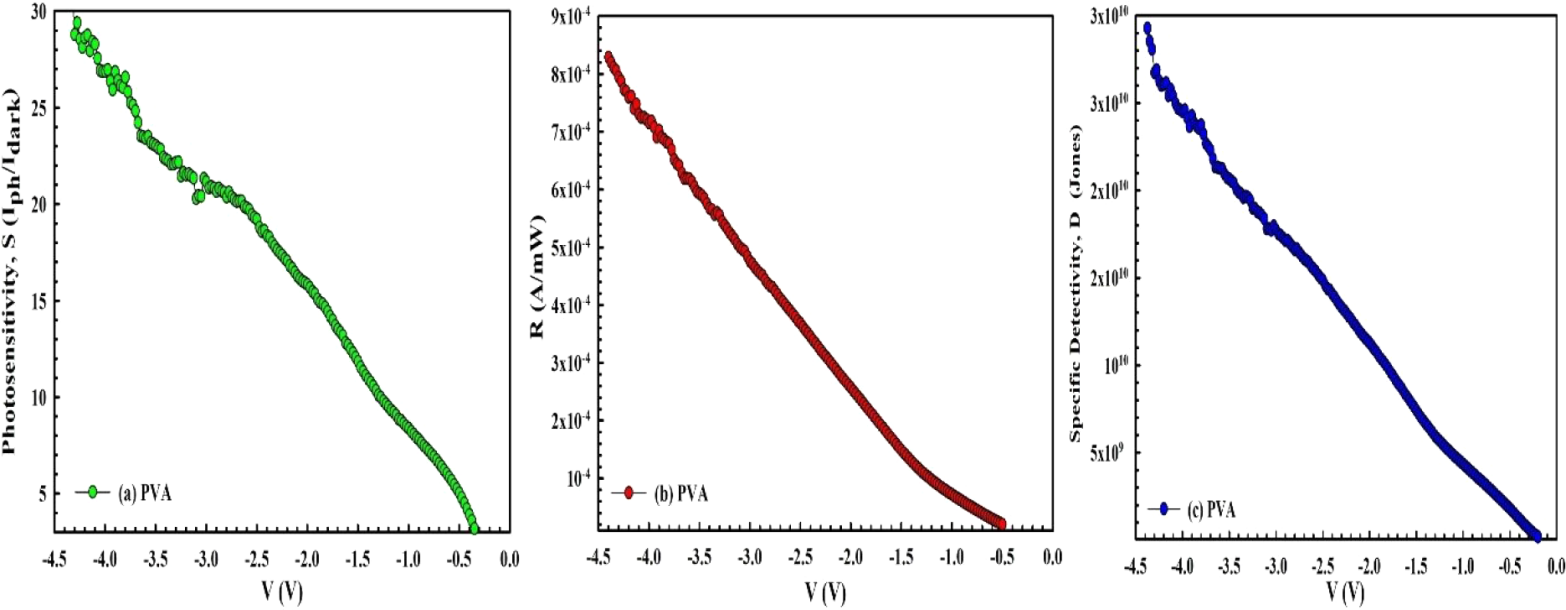
(a) *S* vs *V*, (b) *R* vs *V*, and (c) D* vs *V* plot of the *MPS1*-type *SBD* for various voltages.

**Figure 8:**
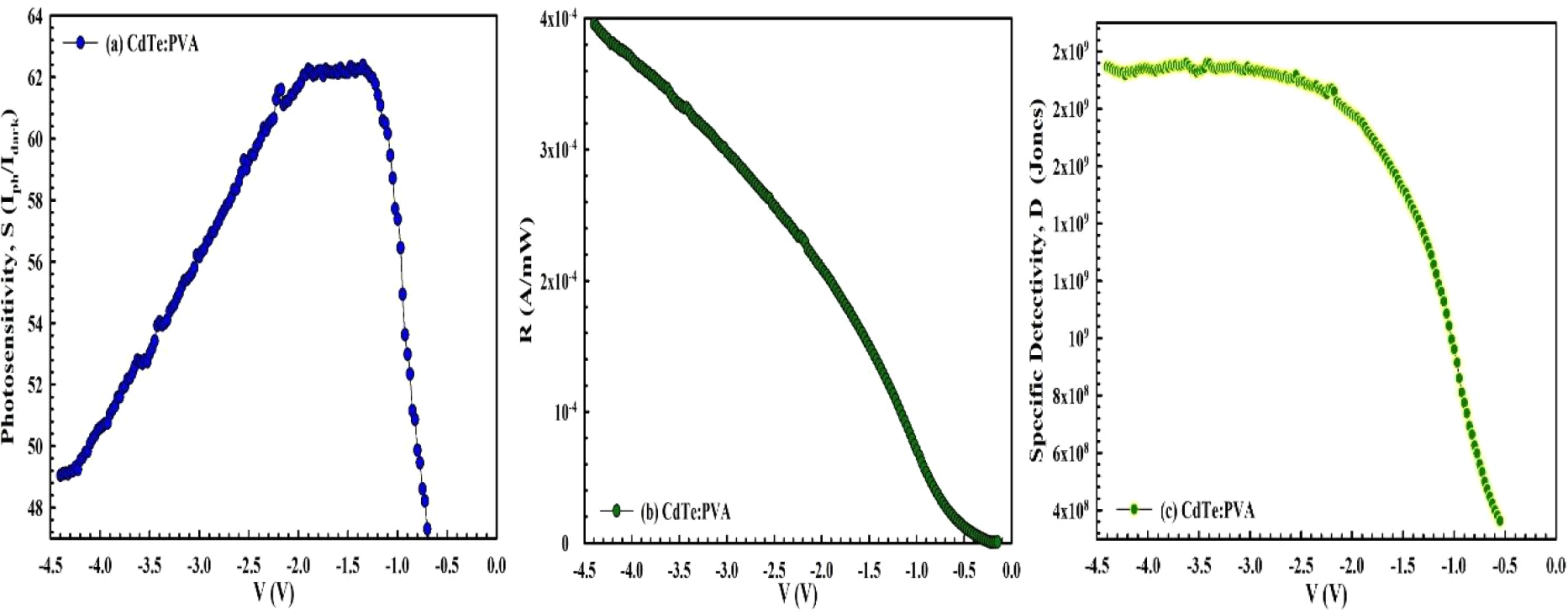
(a) *S* vs *V*, (b) *R* vs *V*, and (c) D* vs *V* plot of the *MPS2*-type *SBD* for various voltages.


9 $$S\left(V\right)=\frac{I_{ph}-I_{d}}{I_{d}}$$

10 $$R\left(V\right)=\frac{I_{ph}-I_{d}}{P^{*}A}$$

11 $$\left(D^{*}\right)=\frac{R\cdot \sqrt{A}}{{\left(2qI_{d}\right)}^{0.5}}$$

As can be seen in both Figure 7a–c and Figure 8a–c, the *S, R,* and D* values are sensitive to light for the two types of *PDs,* and they increase with an increase in voltage in the negative direction for *MPS1* due to the increasing electric field. On the other hand, for *MPS2*, the *S–V* plot has a distinctive peak at around −1.5 V. In addition, as can be seen in Figure 8a–c, the use of 0.05 CdTe-doped PVA leads to a considerable increase in the *S, R,* and D* values. For example, the *S* value of CdTe-doped PVA increased by approximately 2 times when compared with pure PVA.

To determine the current transport mechanisms, the ln­(*I*) vs ln­(*V*) plot for the *MPS1-* and *MPS2*-type *PDs* has been drawn and represented in Figure 9a,b, respectively. As shown in these figures, the ln­(*I*_*F*_) vs ln (*V*_*F*_) plot both in the dark and under illumination, has two linear parts with different slopes, which correspond to moderate and high voltage ranges. The slope of these plots for the first region was found to be 1.638 (in the dark) and 1.244 (under 100 mW/cm^2^) which correspond to space-charge-limited current (*SCLC*) and ohmic behavior, respectively. For the second region, the value of the slope was found to be 2.661 (in the dark) and 3.395 (under 100 mW/cm^2^) which correspond to trap-charge-limited current (*TCLC*). As shown in Figure 9b, the ln­(*I*_F_) vs ln­(*V*_*F*_) plot for Au/CdTe:PVA/n-Si *SBD* also has two different linear parts, like Au/PVA/n-Si *SBD,* with slopes of 5.300 and 3.174, respectively. The *CTM* was governed by *TCLC* for the two linear parts. Similarly, these slopes under illumination conditions were found to be 2.263 and 3.255, respectively. Thus, *CTM* was also governed by *TCLC* under illumination conditions.

**Figure 9:**
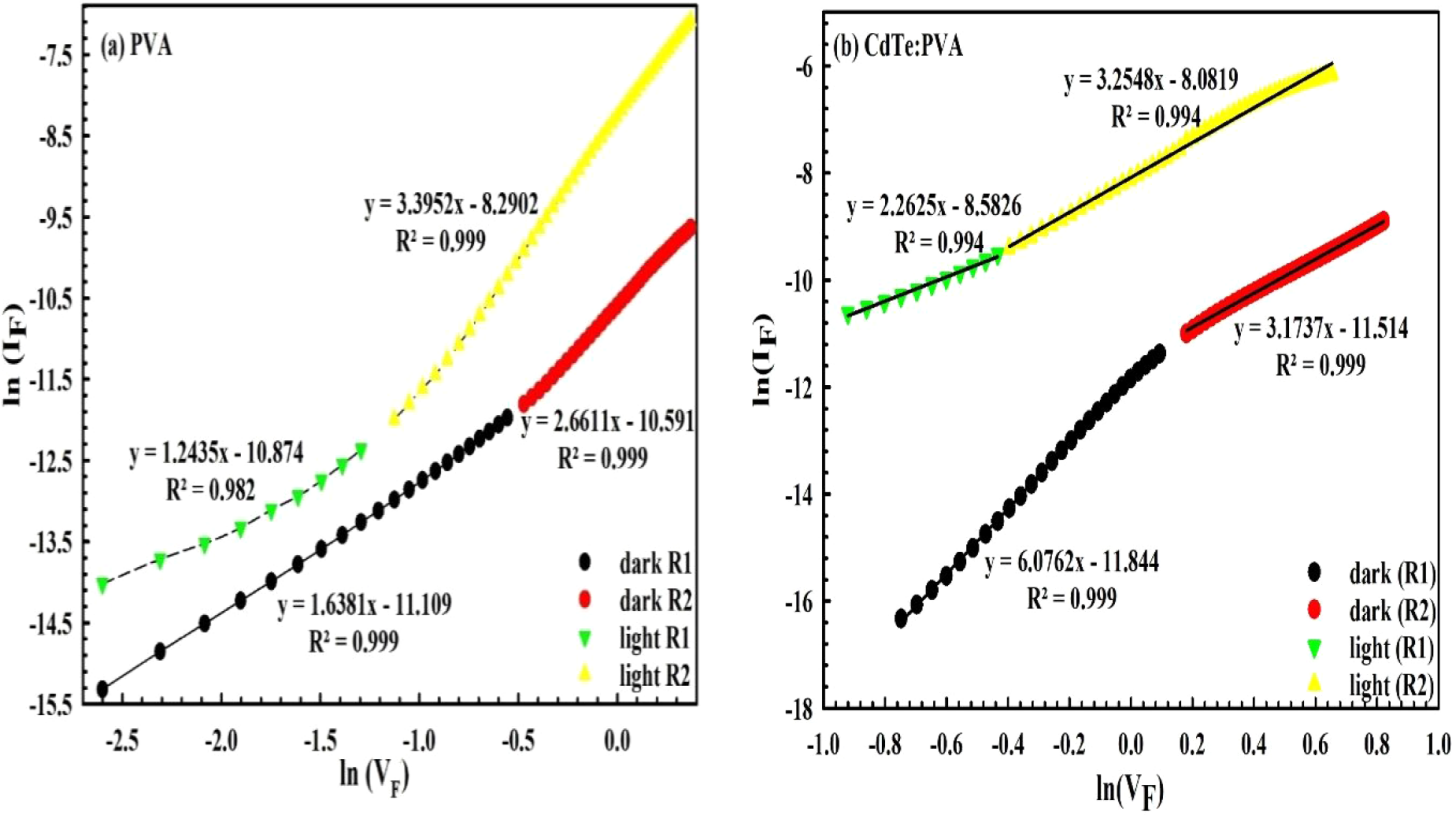
ln­(*I_F_*)–ln­(*V_F_*) curves: (a) for *MPS1* and (b) for *MPS2 PDs* in the dark/illumination.

According to Card and Rhoderick,^5^ when the interlayer width is higher than ∼3 nm, *N*_*ss*_ reaches equilibrium with the semiconductor rather than forming a Schottky contact, and both the *n* and *BH* values become strong functions at positive bias voltages. Thus, the energy density distribution profile of (*N*_***ss***_ vs (*E*_*c*_ *–E*_*ss*_)) in both dark and illumination conditions for these two *SBDs* can be extracted from the *I*_*F*_ and *V*_*F*_ data by considering *V*-dependent ideality factor and *BH,* as demonstrated following eqs 12,13, and 14.^9–15^

12 $$N_{ss\;}\left(V\right)=\frac{\varepsilon_{0}}{q}\left[\frac{\varepsilon_{i}}{d_{i}}\left(n\;\left(V_{i}\right)-1\right)-\frac{\varepsilon_{s}}{W_{D}}\right]$$

13 $$\Phi_{e\;}\left(V\right)={\Phi \;}_{b\;0}+\left(1-\frac{1}{n\left(V\right)}\right)V$$

14 $$q\;\left(E_{c}-E_{ss}\right)=\left({\Phi \;}_{e}-V\right)$$

Thus, the *N*_*SS*_–(*E*_*c*_ *–E*_*ss*_) curves of the *MPS1* and *MPS2* PDs were obtained from the *I*_*F*_ *–V*_*F*_ both in the dark/under 100 mW/cm^2^ and are given in Figure 10a,b, respectively. As shown in Figure 10a,b, the *N*_*ss*_ vs (*E*_*c*_ *–E*_*ss*_) curves for the two *PDs* have an almost similar U-shaped behavior due to the specific distribution of interface traps with energies in the bandgap of Si, which are reordered and restructured under the effects of electric-field illumination.^13–19^

**Figure 10:**
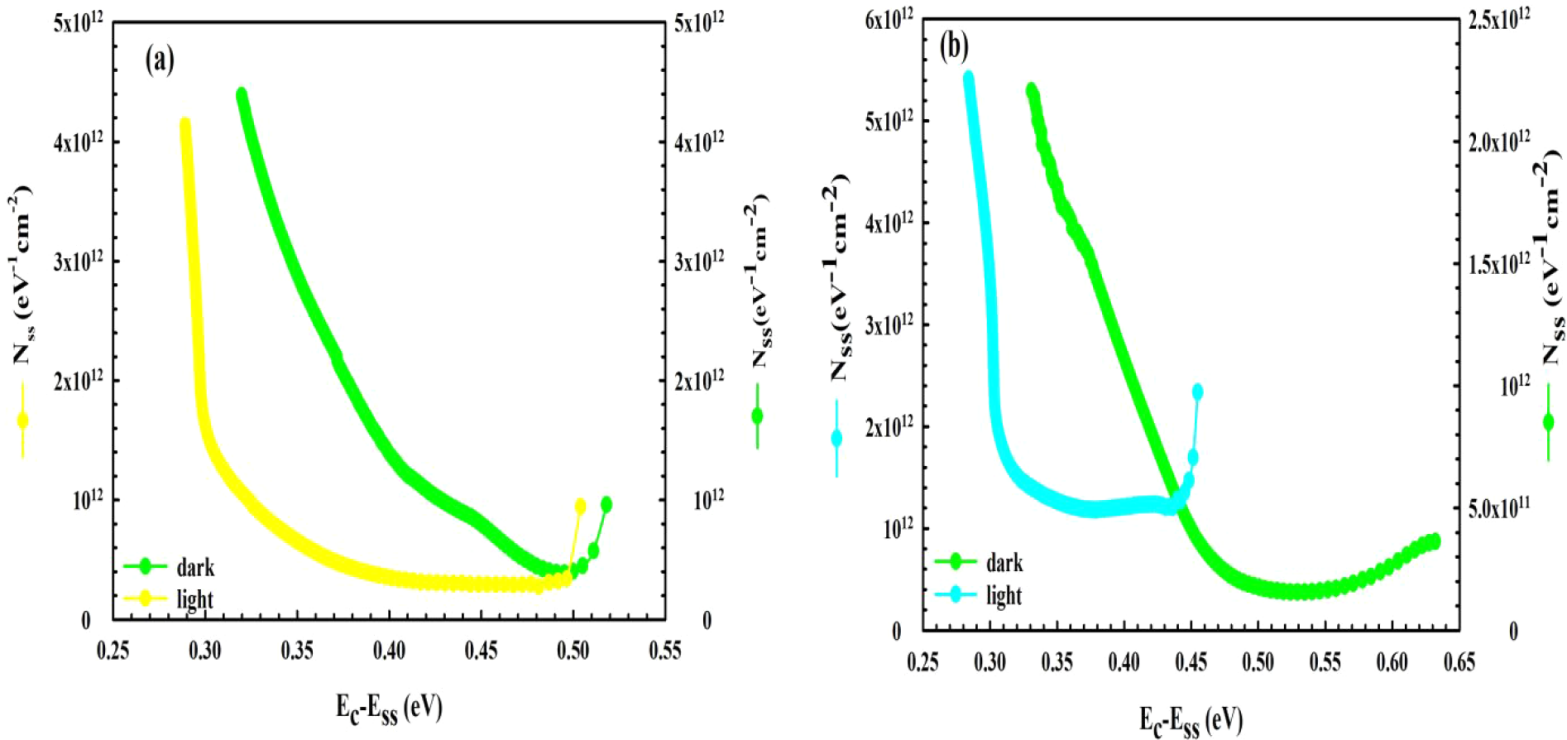
*N_ss_* vs (*E_c_−E_ss_*) curves obtained from the *I_F_–V_F_* data: (a) for *MPS1* and (b) for *MPS2 PDs*, respectively.

## 4 Conclusion

In the present study, both the *MPS1* and *MPS2 PDs* were performed on the *n*-Si wafer by utilizing the spin-coating method to determine the effects of pure/(CdTe:PVA) interfacial layers on the fundamental electrical parameters and conduction mechanisms both in dark and 100 mW/cm^2^ conditions. For this aim, some electric parameters were calculated from the *I–V* measurements and compared with each other in dark and under 100 mW/cm^2^ illumination at *RT* in the voltage range of ±4.5 V. To determine the light sensitivity of the prepared two different *SBDs*, the voltage-dependent profiles *S, R,* and R* were also found under 100 mW/cm^2^ condition and the *S* value of Au/CdTe:PVA/n-Si *SBD* increased by approximately 2 times when compared to pure PVA. Both the energy-dependent profiles of *N*_*ss*_ and voltage-dependent *R*_*i*_ were obtained by using *I–V* characteristics data by using Card-Rhoderick and Ohm’s law methods. The energy-dependent profile of *N*_*ss*_ shows an almost U-shaped behavior due to a special distribution of *N*_*ss*_ at the interlayer/Si interface with energies in the bandgap of Si, and the reordering and restructuring of them under electric field and illumination effects. All experimental findings were found to be strong functions of illumination as well as voltage. However, the *MPS2* photodiode shows good performance and photosensitivity compared to the *MPS1* photodiode.

## Data Availability

All data supporting the conclusions of this study are presented within the article. Supplementary data sets produced or analyzed during the research are available from the corresponding author upon reasonable request.

## References

[1] Sze, S. M. ; Li, Y. ; Ng, K. K. Physics of Semiconductor devices, 4th ed. ed.; Wiley, 2021.

[2] Kanbur H., Altındal Ş., Mammadov T., Şafak Y. (2011). Effects of
illumination on I-V, C-V and G/w-V characteristics of Au/n-CdTe Schottky
barrier diodes. J. Optoelectron. Adv. Mater..

[3] Sharma, B. L. Metal-semiconductor Schottky Barrier Junctions and Their Applications; Plenum Press: New York, 1984.

[4] Rhoderick, E. H. ; Williams, R. H. Metal-Semiconductor Contacts, 2nd ed.; Oxford: Clarendon, 1988.

[5] Card H. C., Rhoderick E. H. (1971). Studies of tunnel MOS diodes I. Interface
effects in
silicon Schottky diodes. J. Phys. D: Appl. Phys..

[6] Yerişkin S. A., Balbaşı M., Orak İ. (2017). The effects of (graphene doped-PVA)
interlayer on the determinative electrical parameters of the Au/n-Si
(MS) structures at room temperature. J. Mater.
Sci: Mater. Electron..

[7] Farazin J., Asl M. S., Pirgholi-Givi G., Delbari S. A., Namini A. S., Altındal Ş., Azizian- Kalandaragh Y. (2021). Effect of
(Co–TeO_2_-doped polyvinylpyrrolidone) organic interlayer
on the electrophysical characteristics of Al/p-Si (MS) structures. J. Mater. Sci.: Mater. Electron..

[8] Barkhordari A., Özçelik S., Altındal Ş., Pirgholi-Givi G., Mashayekhi H., Azizian-Kalandaragh Y. (2021). The effect of PVP: BaTiO_3_ interlayer on
the conduction mechanism and electrical properties at MPS structures. Phys. Scr..

[9] Demirezen S., Altındal Ş., Azizian-Kalandaragh Y., Akbaş A. M. (2022). A comparison of Au/n-Si Schottky diodes (SDs) with/without
a nanographite (NG) interfacial layer by considering interlayer, surface
states (N_ss_) and series resistance (R_s_) effects. Phys. Scr..

[10] Çetinkaya H. G., Demirezen S., Altındal Yerişkin S. (2021). Electrical
parameters of Au/(%1Ni-PVA)/n-Si (MPS) structure: Surface states and
their lifetimes. Phys. B.

[11] Tung R. T. (2014). The physics
and chemistry of the Schottky barrier height. Appl. Phys. Rev..

[12] El-Shamy A. G. (2021). New nano-composite
based on carbon dots (CDots) decorated magnesium oxide (MgO) nano-particles
(CDots@MgO) sensor for high H_2_S gas sensitivity performance. Sens. Actuators, B.

[13] Sasikumar K., Bharathikannan R., Raja M. (2019). Effect of Annealing Temperature on
Structural and Electrical Properties of Al/ZrO_2_/p-Si MIS
Schottky Diodes. Silicon.

[14] Reddy V. R., Prasad C. V. (2018). Surface chemical
states, electrical and carrier transport
properties of Au/ZrO_2_/n-GaN MIS junction with a high-k
ZrO_2_ as an insulating layer. Mater.
Sci. Eng. B.

[15] Baştuğ A., Khalkhali A., Sarıtaş S., Yıldırım M., Güçlü Ç. Ş., Altındal Ş. (2025). Electrical
properties, conduction mechanisms, and voltage dependent curves of
interface traps, series resistance in Au/(Sn: Fe_2_O_3_)/n-Si structures using impedance measurements. J. Mater. Sci.: Mater. Electron..

[16] Sharma M., Tripathi S. K. (2013). Analysis of interface states and
series resistance
for Al/PVA: n-CdS nanocomposite metal–semiconductor and metal–insulator–semiconductor
diode structures. Appl. Phys. A.

[17] Altındal Ş., Sevgili Ö., Azizian-Kalandaragh Y. (2019). A comparison
of electrical parameters of Au/n-Si and Au/(CoSO_4_–PVP)/n-Si
structures (SBDs) to determine the effect of (CoSO_4_–PVP)
organic interlayer at room temperature. J. Mater.
Sci.: Mater. Electron..

[18] Badali Y., Azizian-Kalandaragh Y., Uslu İ., Altındal Ş. (2020). Investigation
of the effect of different Bi_2_O_3_–*x*: PVA (*x* = Sm, Sn, Mo) thin
insulator interface-layer materials on diode parameters. J. Mater. Sci.: Mater. Electron..

[19] Büyükbaş-Uluşan A., Tataroğlu A., Altındal Yerişkin S. (2023). Analysis of
the Current Transport Characteristics (CTCs) in the Au/n-Si Schottky
Diodes (SDs) with Al_2_O_3_ Interfacial Layer over
Wide Temperature Range. ECS J. Solid State Sci.
Technol..

[20] Sevgili Ö., Asar Y. Ş., Altındal Ş., Ulusoy M., Azizian-Kalandaragh Y. (2025). On comparison
of Au/n-Si (MS) Schottky
diodes with and without (Brushite + Monetite: PVC) an
interlayer grown by spin coating technique. Electr. Eng..

[21] Altındal Ş., Azizian-Kalandaragh Y., Ulusoy M., Pirgholi-Givi G. (2022). The illumination
effects on the current conduction mechanisms of the Au/(Er_2_O_3_: PVC)/n-Si (MPS) Schottky diodes. J. Appl. Polym. Sci..

[22] Demirezen S., Ulusoy M., Durmuş H., Çavuşoğlu H., Yılmaz K., Altındal Ş. (2023). Electrical and Photodetector Characteristics
of Schottky Structures Interlaid with P­(EHA) and P­(EHA-*co*-AA) Functional Polymers by the iCVD Method. ACS Omega.

[23] Ata D., Altındal Ş., Balbaşı M. (2025). On an investigation
of optoelectrical features in Au/(MWCNT: PVA-B­(OH)_3_)/n-Si
structure, using I–V data, in dark and illumination conditions. J. Mater. Sci.: Mater. Electron..

[24] Reddy D. S., Kumar A. A., Reddy V. R. (2026). Electrical and photodiode
possessions
of the Au/V_2_O_5_/un-InP MIS-type photodiode under
different illumination light intensities. Mater.
Sci. Eng., B.

[25] Di
Bartolomeo A., Intonti K., Peluso L., Di Marco R., Vocca G., Romeo F., Giubileo F., Grillo A., Orhan E. (2025). Metal-semiconductor Schottky diode with Landauer’s formalism. Nano Express.

[26] Beneldjemoui B., Kacha A. H., Mostefaoui M., Yahi A. H., Akkal B., Benamara Z., Anani M. (2025). Study of the Surface Photovoltage
and Photovoltaic Properties of Au/δ-GaN/*n*-GaAs
Schottky Barrier-Based Photodetectors. Phys.
Solid State.

[27] Karataş Ş., Aydın M. G., Özerli H. (2016). Illumination
impact on electrical properties of Ag/0.6 wt% nanographene oxide doped
poly­(vinyl alcohol) nanocomposite/p-Si heterojunction. J. Alloys Compd..

[28] Çavaş M., Yakuphanoğlu F., Karataş Ş. (2017). The
electrical properties of photodiodes
based on nanostructure gallium doped cadmium oxide/p-type silicon
junctions. Indian J. Phys..

[29] Karataş Ş. (2005). Comparison
of electrical parameters of Zn/p-Si and Sn/p-Si Schottky barrier diodes. Solid State Commun..

[30] Seymen H., Berk N., Orak İ., Karataş Ş. (2022). Effect
of illumination intensity on the electrical characteristics of Au//SiO_2_/n-type Si structures with GO and P3C4MT interface layer. J. Mater. Sci.: Mater. Electron..

[31] Barkhordari A., Altındal Ş., Özçelik S., Mashayekhi H. R., Muradov M., Azizian-Kalandaragh Y. (2025). Enhancement
of the Optoelectric and Photovoltaic Responses of Al/PVP: ZnTiO_3_/p-Si Structure by Graphene Nanoparticles. Adv. Photonics Res..

[32] Altındal Ş., Barkhordari A., Özçelik S., Pirgholi-Givi G., Mashayekhi H. R., Azizian-Kalandaragh Y. (2021). A comparison of electrical characteristics
of Au/n-Si (MS) structures with PVC and (PVC: Sm_2_O_3_) polymer interlayer. Phys. Scr..

[33] Mallikarjuna D., Kumar A. A., Reddy V. R., Kaleemulla S., Janardhanam V., Choi C.-J. (2025). Photovoltaic and
Barrier Properties
of Au/n-Ge Schottky Junction Modified by Methylene Blue Organic Dye
Interlayer. J. Inorg. Organomet. Polym. Mater..

[34] Thapaswini P. P., Padma R., Balaram N., Bindu B., Reddy V. R. (2016). Modification
of electrical properties of Au/n-type InP Schottky diode with a high-k
Ba_0.6_Sr_0.4_TiO_3_ interlayer. Superlattices Microstruct..

[35] Daş E., İncekara Ü., Aydoğan Ş. (2021). A comparative
study on electrical characteristics of Ni/n-Si and Ni/p-Si Schottky
diodes with Pinus Sylvestris Resin interfacial layer in dark and under
illumination at room temperature. Opt. Mater..

[36] Cheung S. K., Cheung N. W. (1986). Extraction of Schottky diode parameters
from forward
current-voltage characteristics. Appl. Phys.
Lett..

[37] Norde H. (1979). A modified
forward I-V plot for Schottky diodes with high series resistance. J. Appl. Phys..

[38] Dere A., Coşkun B., Tataroğlu A., Al-Sehemi A. G., Al-Ghamdi A. A., Alateeq H. M. A., Qindeel R., Farooq W. A., YakuphanoĞlu F. (2018). Boron doped
graphene based linear dynamic range photodiode. Phys. B.

[39] Yıldız K., Yerişkin S. A., Khalkhali A., Dere A., Yakuphanoğlu F. (2025). Photonic sensor
based quaternary metal oxide semiconductor by controlling of solar
photon intensity. Sens. Actuators, A.

[40] Büyükbaş-Uluşan A., Turan R., Altındal Ş. (2025). On the investigation of current transport
mechanisms (CTMs) of the crystalline Si solar cells utilizing current/voltage
(I–V) characteristics in temperature range of 110–380K. J. Mater. Sci.: Mater. Electron..

[41] Wu D., Zhao Z., Lu W., Rogée L., Zeng L., Lin P., Shi Z., Tian Y., Li X., Tsang Y. H. (2021). Highly sensitive solar-blind deep ultraviolet photodetector
based on graphene/PtSe_2_/β-Ga_2_O_3_ 2D/3D Schottky junction with ultrafast speed. Nano Res..

[42] Wu E., Wu D., Jia C., Wang Y., Yuan H., Zeng L., Xu T., Shi Z., Tian Y., Li X. (2019). In Situ Fabrication
of 2D WS_2_/Si Type-II Heterojunction for Self-Powered Broadband
Photodetector with Response up to Mid-Infrared. ACS Photonics.

